# A Review of the Role of Built Environment and Temperature in the Development of Childhood Obesity

**DOI:** 10.7759/cureus.49657

**Published:** 2023-11-29

**Authors:** Atika Jabeen, Muhammad Shuaib Afzal, Sameer A Pathan

**Affiliations:** 1 Public Health, London School of Hygiene & Tropical Medicine, University of London, London, GBR; 2 Emergency Department, Hamad Medical Corporation, Doha, QAT; 3 Emergency Medicine, Blizard Institute, Queen Mary University of London, London, GBR; 4 Emergency Medicine, School of Public Health and Preventive Medicine, Monash University, Melbourne, AUS

**Keywords:** risk factors childhood obesity, childhood obesity, temperature, built environment, environment, paediatric, childhood, obesity

## Abstract

The burden of obesity is rising globally and is studied widely, yet the evidence for the association of environmental factors (both built and natural) with childhood obesity remains inconsistent. A relation with temperature as a proxy for natural environmental factors for obesity has not been reviewed previously. The purpose of this review was to assimilate updated evidence on environmental factors of childhood obesity. Three databases, MEDLINE (Medical Literature Analysis and Retrieval System Online), Web of Science, and Cochrane, were searched for articles related to the effect of built environment and temperature on childhood obesity in 6-12-year-olds published in the last five years. Twelve studies were identified: four longitudinal and eight cross-sectional. The studies were appraised using the National Institute of Health Quality (NIH) Assessment Tool. A review of included studies showed that built environmental features like higher residential and population density, higher intersection density, more playgrounds, and all park features like the presence or availability of parks, high number of parks, proximity to parks, and an increased park land area, showed a protective association against childhood obesity while land use mix showed a promoting association for the development of childhood obesity. Inconclusive evidence was observed for other built environmental features. The search strategy did not retrieve any literature published in the past five years studying the association between temperature and the development of childhood obesity. Standardization of definitions of exposure and outcome measures is recommended. Further research studying the relationship between environmental temperature and the development of childhood obesity is recommended.

## Introduction and background

Obesity is emerging as a global challenge, as its burden is rising alarmingly in both high and low-income countries [1-8]. It is associated with adverse health consequences, including but not limited to the increased burden of non-communicable diseases (NCDs) and chronic diseases, poor morbidity outcomes, and a decreased life expectancy of up to 20 years [1-4,6,7,9-12]. These adverse effects not only impact children’s current health but also their adolescence and adulthood [1-3,10], making early obesity prevention efforts a crucial priority [1].

Along with the efforts to combat obesity at the individual level with dietary modifications and exercise, due attention is being paid to the wider and potentially modifiable determinants of the environment to facilitate individual-level efforts [10,13]. This coincides with the rapid growth of literature after 2005 with an emphasis on community-level aspects [14]. The restructuring of the built environment to create a physical activity-promoting environment aims to achieve a beneficial impact on health outcomes, particularly on obesity outcomes in children. Since children spend a substantial amount of time within their home neighbourhood, it is believed that this approach may support efforts to address the obesity epidemic, although evidence to date has been inconsistent [3,4,10].

The built environment is characterized by features of the urban built environment (intersections, sidewalks, accessibility to public transport, etc.), recreational built environment (outdoor or indoor public gyms, swimming pools, playgrounds, etc.), and greenness (open spaces, parks, etc.). It is hypothesized that child-unfriendly features of the built environment discourage physical activity like walking, running, and cycling, thereby leading to higher childhood obesity rates [4,11,14].

Evidence shows that inclement weather is considered an obstacle to children’s physical activity [15]. For every 10-degree Fahrenheit additional heating, there is a reduction of five-minute daily moderate-vigorous physical activity (MVPA), while for every 10-degree additional cooling (degrees ≥ 65°F), there is a 17-minute MVPA reduction [16]. Since the direct association of temperature with obesity is understudied, it is postulated that inclement or extreme weather conditions may contribute to obesity by discouraging physical activity [14,17]. To avoid unfavourable weather, subsequently, sedentary lifestyle modifications occur such as remaining indoors and indulging in increased screen time for example, or an increased reliance on vehicular transport instead of walking or cycling for a commute. Furthermore, increasing traffic on roads has contributed to global warming, pollution, and expanding complex road networks that in turn may reduce pedestrian or bicycle transport due to safety concerns and unfavourable weather. This could result in increased use of motor transport and lower levels of physical activity leading to a vicious cycle in which both the environment and obesity are conducive [11,14]. The natural environment-built environment relationship is understudied. It is not fully understood how these environmental exposures interact with each other, and how they confound or mediate obesity outcomes. Temperature is considered a significant weather indicator of the natural environment as determined by another review [17], focusing solely on natural environmental factors affecting obesity. The role of the interlinkage between temperature and the built environment in aggravating obesity may thus be a cause of concern and holds potential to study.

**Figure 1:**
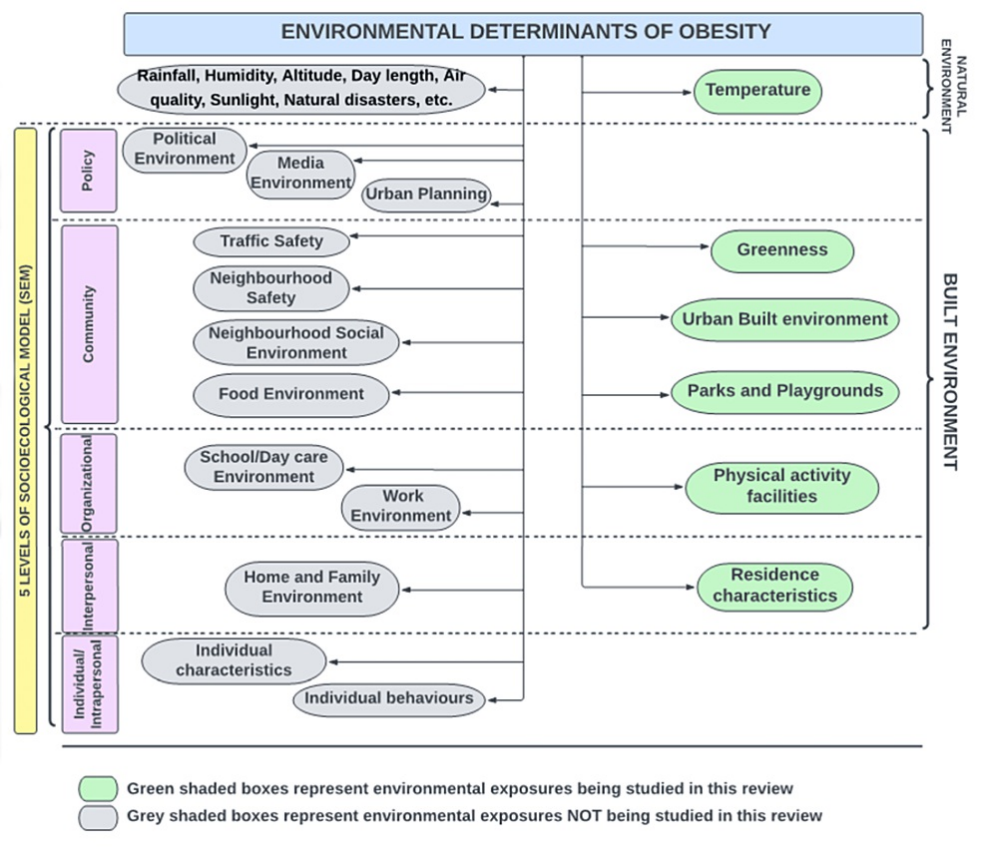
Environmental Determinants of Obesity Image credit: Author Note: This framework, although universal, has been written in the context of this review. There exists an overlap of the different factors within multiple layers of the socioeconomical model, depicted here.

Interactions between various environmental factors (both built and natural) and childhood obesity are interlinked and complex [1]. To better understand them, in 1988, Mc Leroy and colleagues described the socioecological model (SEM) [18] based on five levels: (i) Individual or intrapersonal, (ii) interpersonal, (iii) organizational, (iv) community, and (v) public policy level [11,19]. We generated a conceptual framework based on previously published work to focus review on environmental factors and the SEM levels at which they play a role (Figure 1). Though universal, it has been produced within the context of this review. Nevertheless, it can be modified to expand upon the different categories according to the needs of future research.

Some recent reviews studying the effect of the built environment on obesity were identified [2,3,10,20-26]. Moreover, some outdated reviews exist on the topic [1,11,27-30]. Regarding the natural environment, only one review was identified but it did not study the association of temperature directly with obesity [17]. None of the reviews mentioned above studied the effect of built environment or natural environment together on childhood obesity; therefore, this review was undertaken to study the effect of both these crucial factors on childhood obesity.

Study aims and objectives

**Figure 2:**
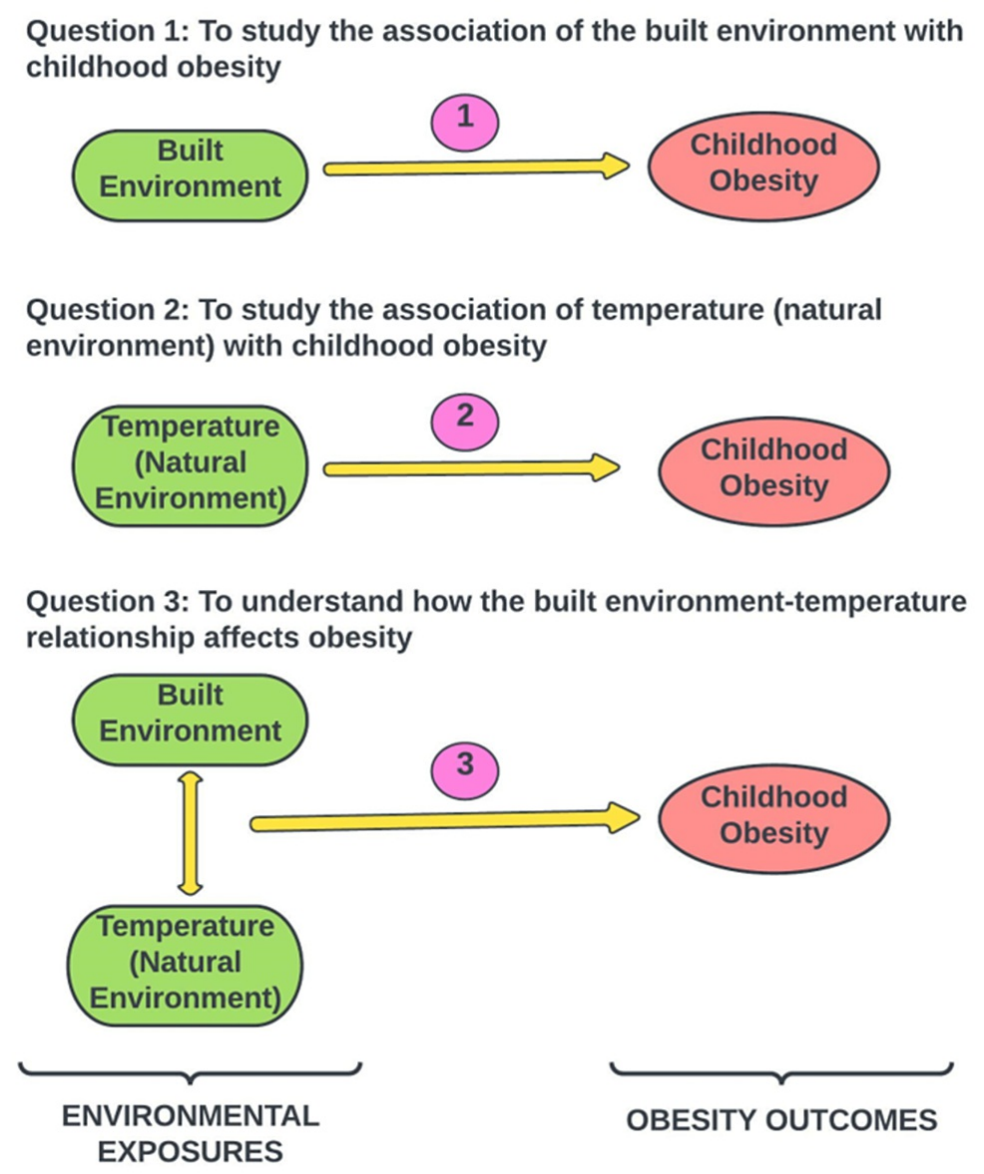
Three main questions of the review Image credit: Author

This study aimed to systematically review recent literature investigating associations between environmental factors (built and natural) and obesity in children between 6-12 years of age around the world. The three main objectives of this review (Figure 2) were: (i) To study the association of the built environment (urban built environment, recreational built environment, greenness) with childhood obesity; (ii) To study the association of temperature (proxy or key determinant of the natural environment) with childhood obesity; (iii) To understand how the built environment-temperature relationship affects the development of childhood obesity. The findings of the review will contribute to future research and aid in urban planning and policymaking.

## Review

The review was assessed by the Research Governance and Integrity Office of the London School of Hygiene and Tropical Medicine as not requiring ethical approval (ethics ref: 26799).

Methods

Eligibility Criteria

The pre-defined eligibility criteria were based on the PICOST (Population, Intervention/Exposure, Comparison group, Outcome, Study design, Time frame) framework. 

The inclusion criteria were: (i) Children between 6-12 years of age belonging to all countries and populations except high-risk obesity populations; (ii) Exposure to the built environment and/or temperature (a key proxy for the natural environment), (iii) Childhood overweight and obesity as the outcome of interest, (iv) Peer-reviewed, primary quantitative research, and (v) Research published from 2017 up to June 2022. The exclusion criteria include: (i) Languages other than English, (ii) Qualitative study designs and reviews, (iii) Age group other than the defined age group as it was hypothesized that an age range below this would be too dependent on parent/caregivers for physical activity-related activities, and an age group above it could be less affected by immediate neighbourhood.

**Figure 3:**
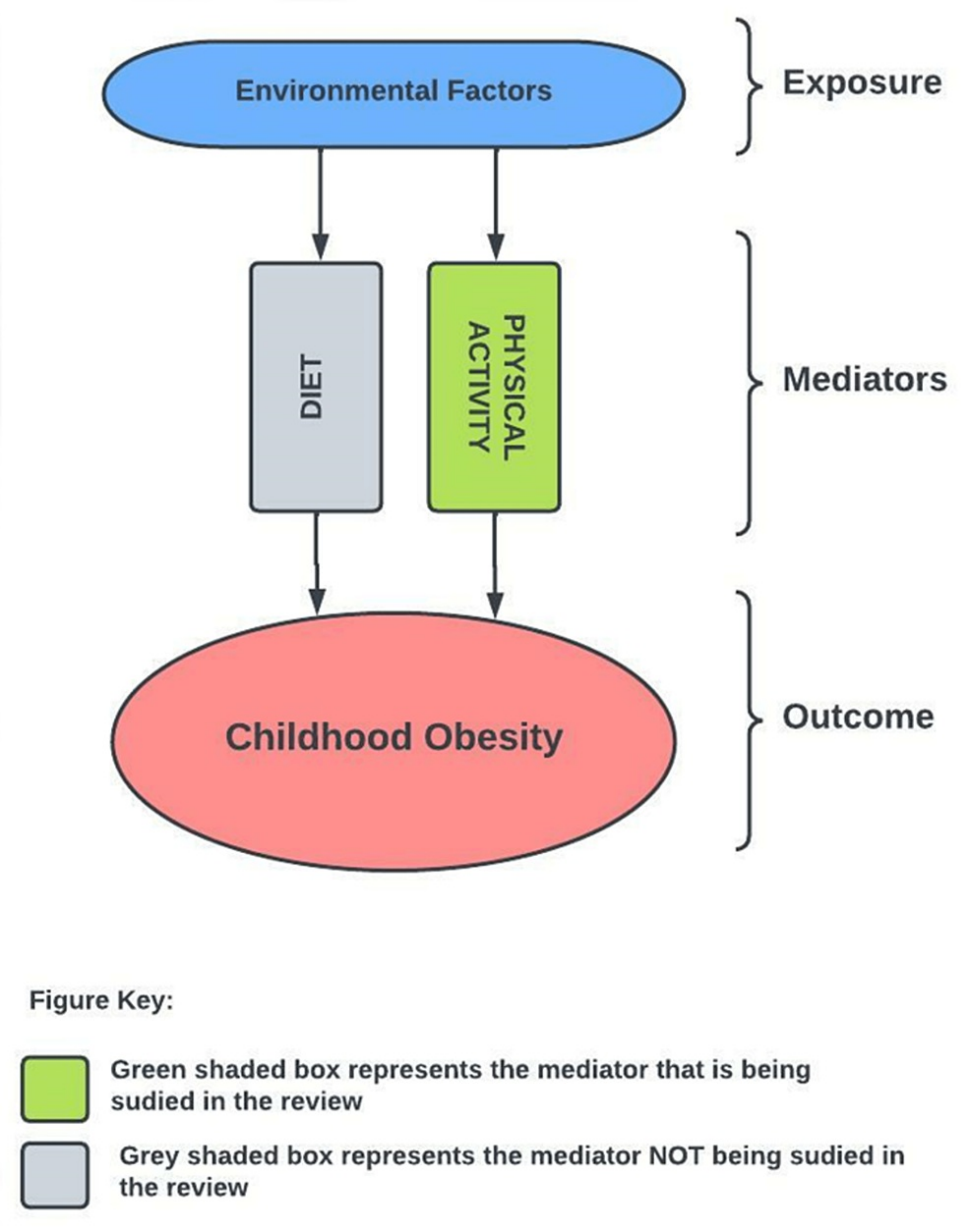
Mediational model for environment-obesity relationship Image credit: Author

While the age range was defined as 6-12 years. it was decided prior to the screening process that an age of ± one year would be acceptable to include as long as most of the age limit fell within the age range of 6-12 years. For example, a study with an age range of 8-13 would be included, but that of an age range of 12-17 would be excluded. Environmental features eligible to study in this review are outlined in Figure 1. As the design and purpose of residential neighbourhoods differ from organizational neighbourhoods such as schools and daycare or work neighbourhoods, it was decided to focus on residential neighbourhoods alone. In the environment-obesity axis, this review focused on only physical activity as the mediator of the relationship, and not dietary factors (Figure 3). Therefore, in articles discussing both, only the physical activities-related data was included in the review. This is because both physical activity and dietary have their own unique mechanisms leading to the development of obesity and this review focused on physical activity-related factors as the primary concern related to childhood obesity.

Information Sources and Search Strategy

Three databases were identified as relevant to the topic: MEDLINE (Medical Literature Analysis and Retrieval System Online) using Ovid, Web of Science using Clarivate, and Cochrane using the Wiley-Blackwell interface. The terms ‘natural environment’, ‘temperature’, ‘built environment’, ‘overweight’, ‘obesity’, and ‘physical activity’ and various combinations of these terms were included in the search strategy (Appendix A).

Study Selection and Data Extraction

Two reviewers (AJ and MSA) independently screened articles for eligibility and reviewed the included studies for data extraction. No disagreements were found. The Rayyan software [31] was used for the screening process. It aided the exclusion of reviews by default red-flagging of words like ‘review’ and ‘systematic’ review in the abstract. A standardized data extraction sheet was developed in Microsoft Excel for Mac (2019) version 16.63.1 (Microsoft corporation, Redmond, Washington, United States). Detailed methods are provided in the Appendix B. 

A total of 1046 articles were retrieved by our search. Sixty-four duplicates were removed, leaving a total of 982 articles. After title and abstract screening, 22 potential articles remained for full texts screening. Of these, 11 articles were excluded as they did not conform to the PICOST eligibility criteria [32-42]. Reasons for exclusion are provided within the detailed methods in the supplementary material, Appendix B. With the addition of one article [43] found through random snowballing from relevant articles, a total of 12 articles were included in the final review [43-54]. Note that Kimberly Daniels authored two different studies both published in 2021. One is a longitudinal study and the other cross-sectional. Both are included in this review. The search did not find any literature related to temperature and also conforming to the eligibility criteria in the past 5 years, therefore the search was extended to the past 10 years to search for potential articles related to temperature.

As the variables studied varied widely, the exposure types were divided into themes and the studies were aggregated based on the types of exposure measurement. This was not an a-priori decision, rather it emerged during the evidence synthesis phase.

Assessing Quality of Evidence

National Heart, Lung and Blood Institute (NHLBI), National Institutes of Health (NIH) Quality Assessment Tool for Observational Cohort and Cross-Sectional Studies [55] was used for critical appraisal of included studies based on previous research [17,25,26] in conjunction with the guidance for assessing the quality of studies. Studies meeting the quality criteria were given a score of 1, whereas those not meeting the criteria or otherwise (Cannot Determine: CD, Not Reported: NR, Not Applicable: NA) were given a score of 0. Responses were summed to get an objective score from 0 to 14.

Results

Included Studies

Twelve articles were included in this review. All included articles discussed the association of the built environment with obesity [43-54].

With regards to the temperature arm of the review, no literature was found in the past five years that studied the relation of the natural environment (temperature) with childhood (6-12 years) obesity. Seven articles of potential relevance were considered [17,56-61]. Of these, five were excluded on title screening [56-60]; one studied the association of weather conditions in adults [56], two studied the association of weather with physical activity and not with obesity [57, 58], the outcome was blood pressure in the fourth [59], and in the fifth, the outcome was mental well-being [60]. The full text was read of the remaining two. Of these, one was excluded as it did not study childhood obesity as the outcome [61] and the last was a review article and was therefore excluded [17]. The articles included in this review article by Jia et al., which scanned the literature from the inception of databases till December 31, 2018, were also studied for potential inclusion and six articles were identified [17]. None of these had been published in the last five years (2017-2022). Three were published in the last 10 years and the remaining even earlier. Of the three in the past 10 years, only two articles studied weather conditions, including temperature [16,62] as the exposure and the outcome was physical activity, not obesity. The new scoping search extended to the past 10 years and identified 10 articles of potential relevance. In three, the population was adults, not children [63-65]. Whereas in six articles, the outcome was not obesity [66-69]. In the last article, neither the population coincided with the childhood age range, nor obesity was the studied outcome [70]. Hence, no eligible literature was found even in the extended search for the past 10 years. In conclusion, as per the search strategy, no literature was found in the past five years that studied the relationship between the natural environment (temperature) and childhood (6-12 years) obesity.

**Figure 4:**
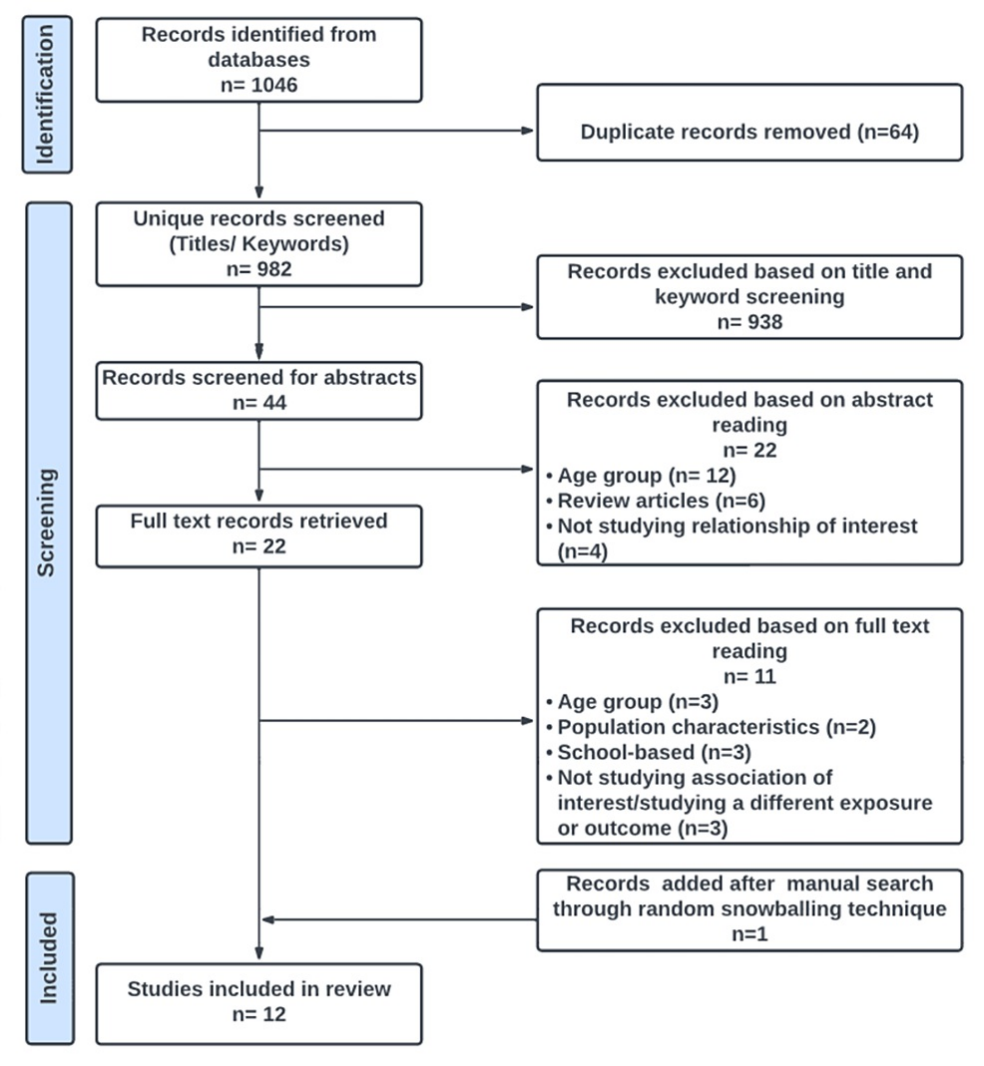
: PRISMA flow diagram for inclusion and exclusion of studies in the review PRISMA: Preferred Reporting Items for Systematic Reviews and Meta-Analyses

The summary of inclusion and exclusion of studies is presented in the Preferred Reporting Items for Systematic Reviews and Meta-Analyses (PRISMA) flow diagram (Figure 4).

Study Characteristics 

Four out of 12 studies were longitudinal by design [43,45,48,53], while the remaining eight were cross-sectional [44,46,47,49-52,54]. The quality assessment using the NIH Quality Assessment Tool for individual study is presented in Table 1. Overall quality scores ranged from 7 to 12.

**Table 1:** Quality assessment of studies included using NIH Quality Assessment Tool for Observational Cohort and Cross-Sectional Studies NR: Not Reported; NA: Not Applicable; CD: Cannot Determine

Study Type		Longitudinal				Cross-sectional								
Author		van der Zwaard et al. (2018) [43]	Daniels et al. (2021) [45]	Jia et al. (2019) [48]	White et al. (2021) [53]	Carlson et al. (2021) [44]	Daniels et al. (2021) [46]	de Bont et al. (2021) [47]	McCarthy et al. (2017) [49]	Molina-Garcia et al. (2021) [50]	Morgan Hughey et al. (2017) [51]	Thielman et al. (2019) [52]	Yang et al. (2017) [54]	
No.	Quality Criteria		Quality Score											
1	Clear research question/ objectives	1	1	1	1	1	1	1	1	1	1	1	1	
2	Clearly defined study population	1	1	1	1	1	1	1	1	1	1	1	1	
3	Participation rate of eligible population	1	1	1	1	1	1	1	1	0	1	1	1	
4	Subject recruitment	1	1	1	1	1	1	1	1	1	1	1	1	
5	Sample size justification	1	1	1	NR	1	1	0	1	0	1	1	1	
6	Measurement of exposure prior to outcome	CD	CD	1	CD	0	0	0	0	0	0	0	0	
7	Time frame sufficiency	1	1	1	1	0	0	0	0	0	0	0	0	
8	Levels of exposure	1	1	1	1	1	1	1	1	1	1	1	1	
9	Exposure measures	1	1	1	1	1	1	1	1	1	1	1	1	
10	Repeated exposure(s)	1	1	1	1	0	0	0	0	0	0	0	0	
11	Outcome measures	1	1	1	1	1	1	1	1	1	1	1	1	
12	Blinding	NR	NR	NR	NR	NR	NR	NR	NR	NR	NR	NR	NR	
13	Loss to follow-up after baseline	NR	NR	0	NR	NA	NA	NA	NA	NA	NA	NA	NA	
14	Potential confounders	1	1	1	1	1	1	1	1	1	1	1	1	
	Total score	11	11	12	10	9	9	8	9	7	9	9	9	

All included studies had a well-defined research question and a clearly defined study population [43-54]. Participation rate was greater than 50% among eligible populations in all studies except for one, thus ensuring representation of the target population and minimizing selection bias. In the pilot study by Molina-Garcia et al., it was 36% [50]. Selection bias was addressed in all studies by subjects being recruited in a uniform manner from pre-defined recruitment pool, based on pre-defined eligibility criteria. A crude justification of the sample size was provided by most studies [43-46,48,49,51,52,54]. The statistical power varied among studies with known sample sizes ranging from 83 in a pilot study conducted in Spain [50], to 13,569 in the United States-based studies by McCarthy et al. and Morgan Hughey et al. [49,51].

Whilst four of our 12 included studies analyzed data longitudinally, it is to be noted that the measurement of exposure prior to outcome was determined in just one study [48]. In others, although repeated measurements were taken and a longitudinal analysis was conducted to assess changes in exposure with changes in outcome over time, the exposure was not assessed prior to the outcome, introducing potential for bias. In all longitudinal studies, the time frame was sufficiently suitable to practically detect an association. Varying levels of exposure as appropriate were used in all studies to enable an assessment of trends and/or dose-response relationships. The measurement of exposures was deemed valid as all included studies used objectively measured data reducing potential for measurement bias. Repeated exposure and outcome measurements were taken in all four longitudinal studies strengthening accuracy of findings. Outcomes were valid and measured objectively in all studies.

Biases (observer or performance) could not be eliminated as blinding was not reported by any study. Loss to follow-up was reported in only one longitudinal study by Jia et al., in which attrition bias may have occurred as the attrition rate was significant, i.e., more than 20%. Although it is worthy to note that this study had the longest follow up duration of nine years with a large sample size to start with. Additionally, this bias was addressed in the study by analyzing those that had stayed and those lost to follow up, observing that they did not differ significantly in their characteristics [48].

All studies accounted for potential confounders through adjustment in the statistical analysis including important demographic characteristics, for example, age and sex. In all studies, the cohort or study sample was not free of the outcome of interest as baseline population was recruited that naturally included overweight and obese children as well. To account for this, some studies adjusted for baseline BMI as well [43,45,47,48,51,53]. Some studies additionally assessed for effect modification with important demographic characteristics [43-46,48,49,51-54].

Baseline characteristics of included studies are presented in Table 2.

**Table 2:** Basic characteristics EHR: Electronic Health Record; ECLS-K: Early Child Longitudinal Study - Kindergarten

Study type	Author	Study location	Sample size	Sample age range in years (Mean, SD)	Sample characteristics
LONGITUDINAL	van der Zwaard et al. (2018) [43]	England, United Kingdom	6001	Subgroup age range 5-11	Children from the United Kingdom Millennium Cohort Study (MCS), a longitudinal cohort between 2005-2012
	Daniels et al. (2021) [45]	Philadelphia, Pennsylvania, United States	Total sample of 51,873 6–19-year-olds (subgroup sample size cannot be determined)	Subgroup age range 6-10	EHR data of a 554-bed hospital in an urban paediatric integrated delivery system between 2007-2016
	Jia et al. (2019) [48]	United States (nationally representative data)	6900	Age 6-7 (Mean age 6.2 years at baseline in 1998).	School children from the ECLS-K cohort between 1998- 2007. Follow-up occurred in 2007.
	White et al. (2021) [53]	Durham, North Carolina, USA	8282	5-12 years	Electronic medical records of children presenting for well child visits to “Duke Children’s Primary Care” from 2012-2017
CROSS-SECTIONAL	Carlson et al. (2021) [44]	Kansas City, Missouri, USA	5121	Subgroup age range 6-8	EHR of children who visited “Children’s Mercy Hospital”, a paediatric primary care clinic between June 2013 and June 2016
	Daniels et al. (2021) [46]	Philadelphia, United States	12700 (48% of total sample 6-19-year-olds, 26,460)	Subgroup age range 6-10	EHR data of out-patient primary care visits in an urban paediatric integrated delivery system in 2014
	De Bont et al. (2021) [47]	Sabadell, Spain	2213	9-12 (mean age= 10.8 ± 0.8)	School children within the ECHOCAT (Urban built environment and childhood obesity in Catalonia) and INMA (Infancia y Medio Ambiente, Environment and Childhood) study at 4^th^, 5^th^ and 6^th^ years of primary school between October 2017 and January 2019
	McCarthy et al. (2017) [49]	South-eastern United States	13,469	Mean age= 9.7 ± 0.99	Third to fifth grade school students in 2013
	Molina-Garcia et al. (2021) [50]	Valencia, Spain	83	6-12	School students’ data from BEACH (Built Environment and Active Children) study between February 2018 to May 2018
	Morgan Hughey et al. (2017) [51]	South-eastern United States	13,469	Mean age= 9.7+-0.99	Third to fifth grade school students in 2013
	Thielman et al. (2019) [52]	Canada (nationally representative data)	1759	Subgroup age range 6-11	Children from the 2007-2011 CHMS (Canadian Health Measures Survey) data
	Yang et al. (2017) [54]	Tennessee, United States	12,016	Mean age of subgroup = 7	Elementary school students in Shelby County Schools (SCS), Tennessee during 2014-2015 academic year

Systematic Review

The measures of environmental exposures, obesity outcomes, and the main findings for each study are summarized in Table 3.

**Table 3:** Summary of exposures, outcomes, and key findings BMI: Body Mass Index; CDC: Centres for Disease Control and Prevention; WHO: World Health Organization; NDVI: Normalized Differential Vegetation Index; OR: Odds Ratio; LSOA: Lower-Level Super Output Area; LUM: Land Use Mix; SEI: Shannon’s Evenness Index; BIA: Bioelectrical Impedance Analysis

Study type	Author (Year)	Measures of environmental exposures	Measures of obesity outcomes	Significant findings
LONGITUDINAL	van der Zwaard et al. (2018) [43]	1) Green space quantity (LSOA), 2) Park quantity including gardens (LSOA)	1) BMI (kg/m^2^), 2) Probability (of being overweight)	Protective association of quantity of gardens with BMI (β = -0.023, 95% CI: -0.047, 0.002, P<=0.1) (0.02 to 0.06 kg/m² per decile increase in effect). No association with the probability of being overweight
	Daniels et al. (2021) [45]	1) Recreational (Physical) activity facility density per km^2^ at 400m buffer (sensitivity analysis at 800m buffer) 2) Greenness (NDVI) from -1 to +1 at 500m buffer (sensitivity analysis at 100m buffer)	Modified BMI z-score based on CDC (age and sex standardized) growth curves (kg/m^2^)	Protective association of greenness with obesity (β = -0.017, 95% CI: -0.023, -0.012, p<0.001)
	Jia et al. (2019) [48]	1) Street connectivity/intersection density/ha, 2) Residential density/ha, 3) Recreational facility density (including fitness facility)/km^2^	1) BMI percentile (age sex-specific) based on CDC 2000 growth chart, 2) Obesity risk (OR)	Protective association of intersection density with average BMI percentiles (β=−0.49, SE=0.19, p < 0.01) and overweight and obesity risk (OR=0.79, 95% CI: 0.66, 0.94) Non-linear U-shaped association with residential density with higher quartiles showing protective association, but up to a certain point compared to lower levels
	White et al. (2021) [53]	1) Sidewalks at 400 m buffer, 2) Trails (including Healthy Mile Trails) at 400 m buffer, 3) Park availability at 400 m buffer	BMIp95 (Age and sex specific BMI 95th percentile	Protective association of parks with obesity (β = -2.85, 95% CI: -5.47, -0.24; P = 0.032).
CROSS-SECTIONAL	Carlson et al. (2021) [44]	1) Street connectivity/ Intersection density per mile^2^, 2) Walkability (Index from 1-20; Score from 0-100), 3) Residential density/ acre, 4) LUM (entropy 0 to 1)	1) BMI percentile, 2) BMI z-score	No statistically significant association found
	Daniels et al. (2021) [46]	1) Walkability (Mean walk score per census tract), 2) Greenness (NDVI from -1 to +1)	BMI z-score (age and sex adjusted) (CDC)	No statistically significant association found
	de Bont et al. (2021) [47]	1) Street connectivity/ intersection density/km^2^ at 100 m and 300 m buffers, 2) Accessibility/bus stop density/km^2^ at 100 m, 300 m and 500 m buffers, 3) Walkability (index) from 0 to 1 within 300 m, 4) Population density/km, 5) LUM (SEI) from 0 to 1 within 300 m, 6) Greenness (Presence Y/N; NDVI at 100m, 300m, and 500m buffers), 7) Green space proximity (m), 8) Green space size	1) BMI z-score (WHO), 2) Overweight/ obesity status, 3) Waist-circumference z-scores (Intraabdominal obesity), 4) Body fat % (BIA) for body composition	LUM associated with promotion of obesity (0.2 units increase of land use mix (OR = 1.30, 95% CI: 1.01; 1.69, p-value= 0.05)
	McCarthy et al. (2017) [49]	1) Playground availability (Y/N), 2) Playground quality	BMI percentile (CDC)	Protective association of playground quality with obesity (OR=0.82, 95%CI: 0.66-1.03, p=0.08)
	Molina-Garcia et al. (2021) [50]	1) Playground quantity, 2) Playground proximity (m), 3) Park quantity, 4) Park proximity (m), 5) Park land area (m^2^)	BMI percentile (CDC) (age and sex-adjusted)	Protective association of park land area with obesity in the 250 m and 500 m buffers (but not in the 1000 m and 1250 m buffers)
	Morgan Hughey et al. (2017) [51]	1) Playground quantity, 2) Park quantity	BMI percentile (CDC 2014)	Protective association of playground and park quantity (b= -1.1, p<0.05) for females, but not for males
	Thielman et al. (2019) [52]	1) Walkability (street-smart walk score) from 0-100	BMI z-score (age adjusted) (WHO)	No statistically significant association found
	Yang et al. (2017) [54]	1) Intersection density/mile^2^, 2) Walk score from 0-100, 3) Population density (1000 persons/ mile^2^), 4) Park proximity (mile) population-weighted	1) BMI percentile (CDC 2017), 2) Overweight+ obesity risk (>= 85^th^ percentile), 3) Obesity risk (>= 95^th^ percentile)	Protective association of population density (OR = 0.93, 95%CI: 0.88, 0.99) and park proximity (OR= 1.01. 95%CI= 1.00, 1.01) with obesity

The 21 built environmental exposures under study were broadly categorized into (i) Urban built environment, (ii) Recreational built environment, and (iii) Greenness. Three major themes of associations emerged that were grouped as promoting obesity, protecting against obesity, and found no associations (Table 4).

**Table 4:** Overview of statistically significant associations of environmental exposures with obesity Note: The numbers in association columns indicate the references of included studies. Playgrounds with presence of play equipment were included in the recreational built environment, while parks were included as a measure of greenness.

Theme	Sr. No	Environmental exposures	Positive association (Possibly promotes obesity)	Negative association (Possibly protects against obesity)	No association
Urban Built Environment	1	Intersection density (street connectivity)		[48]	[44,47,54]
	2	Sidewalks			[53]
	3	Accessibility/bus stop density			[47]
	4	Walkability			[44,46,47,52, 54]
	5	Residential density		[48]	[44]
	6	Population density		[54]	[47]
	7	Land use mix	[47]		[44]
Recreational Built Environment (including Playgrounds)	1	Recreational facilities (including fitness facilities)			[45,48]
	2	Trails, healthy mile trails			[53]
	3	Playground availability			[49]
	4	Playground quantity		[51]	[50,51]
	5	Playground proximity			[50]
	6	Playground quality			[49]
Greenness (including Parks)	1	Greenness/green space availability			[45-47]
	2	Green space quantity			[43]
	3	Green space proximity			[47]
	4	Green space size			[47]
	5	Park availability		[53]	
	6	Park quantity (including gardens)		[43,51]	[43,50,51]
	7	Park proximity		[54]	[50]
	8	Park land area		[50]	

There was remarkable heterogeneity in exposure measurement due to the differences in the operationalization of built environmental definitions. For example, walkability is a composite measure that has been operationalized differently in different studies [44,46,47,52,54]. de Bont et al. operationalized the “Walk score” as a measure of the population density, connectivity density, facility richness index and land use deriving a score of 0 to 1 [47]. Thielman et al., Yang et al., and Carlson et al. each used the same “Walk Score” that ranged from 0 to 100. It was based on the number, variety, and proximity of different neighbourhood amenities, street connectivity, and proximity and density of neighbourhood resources [44,52,54]. Carlson et al. additionally used the United States’ national walkability index, a composite measure of street connectivity, land use mix (LUM), and transit access. It is notable that both measures studied by Carlson et al. achieved similar results [44]. Similarly, a variety of buffer sizes were used in various included studies. These were based on local or regional geographic information systems, and based on individual study preferences and estimates of reasonable walking distances by children. As evident in Table 3, although all studies used BMI (either BMI percentiles or z-scores) for obesity measurement, the reference values for BMI cut-offs varied across studies as some used CDC growth charts as reference [45,48-51,53,54], while others used WHO reference values [47,52]. The choice of BMI reference was not reported in others [43,44]. Only one study used body fat composition measures in addition to BMI outcomes [47].

It is to be noted that due to the presence of play equipment, playgrounds were included in the recreational built environment while parks were included as a measure of greenness. “Healthy Mile Trails” were specially built continuous sidewalks with 1-mile markings for the purpose of increasing recreational activity in Durham, United States, thus the distinction from regular trails [53].

Urban Built Environment

Intersection density: A longitudinal study by Jia et al. with nine years follow-up (n=6900) that reported intersection density (street connectivity) showed a protective association against average BMI percentiles (β=−0.49, standard error (SE)=0.19, p < 0.01) and overweight obesity risk (OR=0.79, 95%CI: 0.66, 0.94) [48]. On the other hand, de Bont et al., Yang et al. (OR=1.00, 95%CI: 1.00,1.00), and Carlson et al. observed no significant association with obesity [44,47,54].

Residential density: Patterns of residential density studied by Jia et al. showed non-linear U-shaped curvilinear trends [48]. Such that higher quartiles of residential density compared to lower quartiles showed protection against obesity association for up to nine years, after which the trend showed promoting obesity association. On the contrary, Carlson et al. observed no association of obesity with residential density [44].

Population density: Yang et al. observed that population density was found to be inversely associated with the risk of overweight and obesity (OR = 0.93, 95%CI: 0.88, 0.99) [54]. On the contrary, de Bont et al. did not find any association between population density and obesity outcomes [47].

LUM: de Bont et al. also observed that per 0.2 units increase of LUM was associated with 1.3 times higher odds of overweight/obesity status (95%CI: 1.01; 1.69, p-value= 0.05) after adjusting for confounding between urban exposures [47]. This was inconsistent with Carlson et al.’s findings of no association of LUM with BMI percentiles [44].

Other urban built features: Sidewalks [53], accessibility/bus stop density (β= -0.07, 95%CI: -0.17, 0.03, p=0.05) [47], and walkability [44,46,47,52,54] did not show any association with obesity.

Recreational Built Environment

The quantity (number) of playgrounds was significantly associated with the BMI percentile (b= -1.1, p<0.05), after adjustment of both socioeconomic status and race/ethnicity, in females, but not in males [51]. In the nationally representative United States cohort, Jia et al. found no association of an increase in physical activity facilities (recreational and fitness facilities) to be associated with children’s weight status or with risk of overweight and obesity, after nine years of follow-up [48]. Daniels et al. similarly found no association between recreation facilities and obesity outcomes [45]. However, it should be noted here that due to the lack of availability of longitudinal data, Daniels et al. did not include public recreational facilities (playgrounds or basketball courts) in physical activity facilities. Overall, trails including healthy mile trails [53], playground availability [49], proximity [50], and quality [49] (OR=0.82, 95%CI: 0.66-1.03, p=0.08) did not show an association with obesity outcomes.

Greenness

Park access, determined by their presence or availability, was associated with a significant reduction in BMI percentile (β = -2.85, 95%CI: -5.47, -0.24; P = 0.032) [53]. van der Zwaard et al. studied the effect of the number of parks on two outcomes, BMI and the probability of being overweight [43]. A significant protective association was observed for changes in the number of gardens (park quantity) and changes in BMI (β = -0.023, 95%CI: -0.047, 0.002, P<=0.1), although the size of the effect was small (0.02-0.06 kg/m² per decile increase in effect), whereas no association was found between the number of gardens and the probability of being overweight [43]. Morgan Hughey et al. determined that the association was significant in females after adjustment for ethnicity (b= -2.2, p<0.05); whereas no association was observed when adjusted for socioeconomic status in females or for males (b=1.5, p=0.08) [51]. On the contrary, Molina-Garcia et al. observed that the number of parks was not related to BMI [50]. Yang et al. observed a direct association between distance to the nearest park with risk of overweight and obesity (OR= 1.01. 95%CI= 1.00, 1.01) [54]. Whereas Molina-Garcia et al. observed that the proximity of parks was not related to BMI [50]. Park land area was observed to be inversely related to the BMI percentile for lower buffers (250 m and 500 m), though not for the higher buffer sizes (1000 m or 1250 m buffers) [50]. Availability [45-47], quantity [43], proximity (β = -0.04, 95%CI: -0.14, 0.06) [47], and size of green spaces [47] (β = -0.07, 95%CI: -0.17, 0.03) were not associated with obesity outcomes amongst 6-12 years age group.

Generalizability

As the included studies spanned only four countries, they did not reflect a global representation. Eight were conducted in the United States, one in Canada, two in Spain, and one in the United Kingdom. The longitudinal study by Jia et al. consisted of data from 6900 children nationally representative across the United States; therefore, its results may be generalizable across the country but with caution because of potential selection bias owing to the high attrition rate [48]. Similarly, Thielman et al. studied nationally representative Canadian data and, in addition, respondents were weighted to be nationally representative [52]. The remaining 10 studies were locally based; therefore, their results should be utilized with caution in other contexts unless having similar population characteristics [43-47,49-51,53,54]. In the study by van der Zwaard et al., the analysis was designed to over-represent minority groups in the United Kingdom [43]. Therefore, its results may be generalized to other groups with caution [43]. The studies by Daniels et al. [45] and Yang et al. [54] included a predominantly African American population; therefore, its results could be applied to these populations, although contextual regional variations should still be considered. Due to the lack of any studies in lower-middle-income countries, the results of this review may not be generalized, especially in such countries.

Discussion

The park availability and land area exhibited a protective association against childhood obesity. Most variables manifested mixed associations with obesity outcomes while others observed no association.

The first objective of the review could be addressed as all 12 included articles discussed the association of the built environment with obesity [43-54]. The second and third objectives could not be sufficiently answered as the search strategy did not reveal any literature published in the past five years studying the effect of temperature in the natural environment on childhood obesity (age range of 6-12 years). For this variable (temperature) only, the search was extended to the past 10 years; however, no literature could be identified that directly studied the association of temperature with childhood obesity. Nevertheless, as Miller-Halegoua pointed out, it is imperative to share these null findings as these could have important policy, practice, and research implications [71]. Temperature may have important implications for childhood obesity. This review highlights this crucial gap in the literature, reflecting the need for future primary research to explore the association between environmental temperature and childhood obesity.

Inconclusive results were observed for most studies looking for an association between environmental exposures and childhood obesity. Urban-built features like street intersection density, walkways, public transportation access, and walkability was found not to be associated with obesity in another review by Jia et al. [21]. On the other hand, the review by Wei et al. observed sidewalk accessibility to be negatively associated with obesity [22]. Unlike inconclusive evidence for the association of residential density and obesity observed in this review, a positive association was found in the review by An et al. [26]. Recreational built features and greenness were found to be negatively associated with obesity in the review by Daniels et al., contrary to no associations observed in this review [25]. 

The most obvious reason for heterogeneity in the findings of this review and in other studies was probably the lack of standardized definitions of the built environment, as well as the lack of standardized measures of exposures and outcomes. This was a challenge faced by previous researchers as well, all of whom highlighted a need to address the problem [1,11,25,41,72]. This may have prevented a fair comparison across studies, denying a meaningful interpretation of the results. Despite evidence suggesting that playgrounds are the most important feature of parks, especially for young children, the fact that this review observed no clear protective association with playgrounds is surprising and yet again could be a result of unclear definitions of parks and playgrounds, the lack of separate assessment of playgrounds within parks, and of parks within green spaces. Clear definitions of green spaces distinguishing green spaces within parks, open green spaces from roadside vegetation, and distinguishing natural from man-maintained vegetation will be required to identify actual associations of types of greenness with obesity and to determine the need for active interventions where required.

A plausible explanation of inconsistencies observed could be related to the complexities of obesity-related risk factors as a dense and accessible neighbourhood not only allows access to physical activity resources but also eases access to unhealthy food choices. Moreover, utility patterns may play a role in the actual determination of obesity, e.g., seasonal variations, gender or race-based variations, and safety perceptions affecting the utility of parks, walkways, and physical activity resources. Utilization of physical activity resources including playgrounds in recent years could also have been affected by coronavirus disease 2019 (COVID-19) and its associated lockdowns throughout the world [73].

Obesity is a result of the imbalance between energy input and output [65,74]. In line with our hypothesis, inclement weather reflected predominantly by temperature may be linked to increased obesity through decreased energy expenditure. This may occur through either decreased physical activity [15,16,75,76] or sedentary behavior [65,74]. von Hippel observed a 0.7% increase in obesity prevalence in adults, with a 1 standard deviation (SD) decrease that is 11 degrees F in temperature in January, and a 1 SD increase (5 degrees F) in temperature in July [63]. Wen et al. deduced that in Chinese adults, the change of weather conditions from rainy to sunny days decreased sedentariness by 6.89 minutes and to cloudy days by 5.6 minutes [56]. Similarly, Valdes et al. determined that in adults, there was a positive association between mean annual temperature and odds of obesity [65]. With regards to the joint effect of the built environment-natural environment relationship on obesity, von Hippel pointed out that despite the availability or existence of a favourable built environment features like sidewalks, bicycle paths, parks etc., these could be neglected if unhospitable weather conditions prevailed [63]. 

In contrast to the overwhelming amount of literature related to the built environment, the scarcity of literature relating to the effects of weather particularly of temperature on obesity in children, is surprising, exemplifying the even greater need for future primary studies in children. Jia et al. in their review voiced the same concern as the current review, quoting, “…the association between natural environment and childhood obesity has received too little scholarly attention” [17].

Other relevant potential reasons for obtaining variable results could be, as Jacobs et al. pointed out, the lack of a genuine association or insufficient power to detect differences [36]. Moreover, different study designs could also have led to different results. Although cross-sectional analyses provide useful information regarding trends, etc., the potential for reverse causation may introduce bias. Instead of the environment causing obesity, it may be that individuals self-select into favourable neighbourhoods, potentially jeopardizing the findings of this review as well as of existing literature. The potential for reverse causality may be less likely for exposure to temperature, as it may not be as easy to select the natural environment, requiring migration possibly to a different region and thus climate. Confounding factors like parents’ socioeconomic status or education levels could act as important drivers for residence into neighbourhoods offering a physical activity-supportive infrastructure. Despite adjustment of confounders, residual confounding of unmeasured variables may limit our ability to make causal inferences. To address the issue effectively, prospective study designs in which exposure precedes the outcome, repeated exposure measurements are taken, and residential instability is accounted for, will be most useful as conducted by Jia et al. [48].

There is a debate regarding the right tool for obesity measurement. Overweight is defined as excess body weight, while obesity is defined as excess body fat. So, although BMI is considered a ubiquitous tool allowing comparison across studies, it is unable to distinguish excess body fat from excess muscle or lean mass [77]. Nevertheless, it is a good proxy of obesity in average-built children, who compose the general majority. Measurement of central adiposity is a better predictor of obesity and of the risk of cardiovascular disease [77]. Most of the studies included in the review used electronic health records, and national or local data that included children’s anthropometric measurements. These datasets may or may not have been specifically designed for obesity research. Also, clinician visits may not allow lengthy measurements to be taken. Body fat measurements including bioelectrical impedance analysis (BIA) were taken by only one of the included studies in addition to BMI [47]. This is possibly due to the relative difficulty in measuring body fat in children compared to adults and the expertise required. Further, there is a lack of evidence of the effectiveness of one over the other form of body fat measurement [77]. Future research should aim to specifically design obesity-related studies and study BMI in children, based preferably on multiple references to allow and aid international comparisons, an example of which is the study by Paciencia et al. [38]. It should also attempt to measure body fat in the childhood age group whenever possible. 

There exists an abundance of literature relating environmental factors to physical activity [14,74,75,78-80]. Regardless, it may be questionable to use such literature that does not directly relate to obesity, to infer the effects of the built environment on obesity. This is due to the multi-factorial aetiology of obesity, of which physical activity is but one component; diet is the other major component, among other factors [63]. Moreover, our thoughts resonate with Davison and Lawson’s proposal in reference to the effect of green spaces on physical activity, that the associations in children may be different from that of adults [81]. Therefore, the results of literature studying environmental effects in adults should not be implemented in children by default.

Limitations of the Review

Firstly, only selected databases were searched for literature published since 2017 in order to review high-quality recent data. Because of time constraints, other databases like Scopus and CINAHL (Cumulative Index to Nursing and Allied Health Literature) Complete could not be searched. Nevertheless, important literature was identified. Secondly, although random snowballing was conducted, systematic snowballing of the reference list of reviews was not conducted due to time constraints. Thirdly, there was remarkable heterogeneity in exposure measurement due to the differences in defining built environmental terms. Fourthly, another limitation of the review is insufficient data to detect associations due to few articles studying each exposure, as shown in Table 4. Most studies discussed only one feature that corresponded to our eligibility criteria. “Walkability” was the most studied variable, studied by five authors. Nevertheless, due to the relative scarcity of data related to each exposure, inferences should be implemented with caution. Due to the multitude of built environment features studied, the effect interactions related to each exposure could not be studied in detail. Nevertheless, significant effect interactions have been reported. Fifthly, the limited age frame could be another limitation of the review. As noted earlier, this was an arbitrary choice that seemed an appropriate age group for the study, but there is no consensus on the right age sub-group to be studied. Finally, the review population mainly represents the United States [44-46,48,49,51,53,54], Canada [52], Spain [47,50] and the United Kingdom [43]. Due to the lack of any studies in lower-middle-income countries, the results of this review may not be generalizable to these countries.

Strengths of the Review

The review summarizes the most recent literature on environmental factors (built and natural) and the association with childhood obesity. This is the first review in recent years that sought to study the association between built and natural environmental factors and obesity. While many reviews have explored how gender, sex, age, race, and socioeconomic status confound the association between environmental factors and obesity, none have studied how temperature, which is a key proxy of the natural environment, affects this relationship. Although the synthesis of evidence was not made possible by the lack of literature related to temperature, this review has, nevertheless, highlighted this crucial gap in the literature and the needs for future primary research.

The conceptual framework (Figure 1) provides an effective overview of the topic, that may be particularly useful to novice researchers. Furthermore, it will aid much-needed efforts to standardize the definitions related to the built environment as it includes a comprehensive categorization of the environmental factors. A visual framework by Casey et al. and Lynch’s lexicon does exist [28,82], yet there was a need to produce a simpler framework. Moreover, this proposed framework has the added strength of incorporating the SEM that has been related to the environmental correlates of childhood obesity [11,19,28]. Another strength in this regard is that all included studies objectively measured data for environmental exposures as well as obesity outcomes, which enables the translation of findings into policymaking and implementation.

Finally, this review brought to attention a lack of literature from lower-middle-income countries, also noted by other authors [26]. This is ironic, as these countries face the dual burden of disease with the co-existence of malnutrition and obesity leading to a plethora of health problems [4,5,7,8]. Similarly, significant disparities exist in disadvantaged regions within developed countries [14]. This may affect the generalizability of our results to socioeconomically disadvantaged countries, cautioning contextual implementation of the findings. There is a call for future research in these regions.

Recommendations for Future Research

This review proposes that childhood obesity-related literature standardizes the use of terminologies, definitions, and outcomes reporting. In this regard, a consensus by leading experts in the field is encouraged. Considering climate change and the alarming rise of obesity, future studies should prioritise the study of the association of temperature with obesity, as well as the effects of the “built environment-natural environment” relationship on childhood obesity, to fill this crucial gap in the literature. There is a call for research to be conducted in lower-middle income countries. A consideration of the built environment is crucial in urban design due to the potential obesoprotective effects of a physical activity-supportive environment. Neighbourhood features that confer protection against childhood obesity like intersection density (street connectivity) and the number of playgrounds and parks should be prioritized based on the strength and consistency of evidence in this review.

## Conclusions

This review underscores the complex interplay between environmental factors and childhood obesity. While a protective association was identified concerning park availability and land area, findings across other variables were inconclusive, emphasizing the intricate nature of this relationship. Crucially, a significant research gap exists regarding the influence of temperature, a key natural environmental factor, on childhood obesity. This scarcity of studies highlights the urgent need for comprehensive research in this area, particularly in the context of climate change and shifting physical activity patterns.

Standardizing terminologies and outcomes reporting is essential for meaningful cross-study comparisons. Additionally, understanding the socioecological nuances, such as socioeconomic disparities and seasonal variations, is vital for a comprehensive understanding of childhood obesity. Future research efforts should prioritize investigating the impact of temperature on childhood obesity and focus on diverse socioecological factors. Addressing these challenges will enhance our understanding, inform policies, and guide interventions to effectively tackle childhood obesity globally.

## Appendices

Appendix A: search strategy

**Table 5:** Ovid MEDLINE Search Database: Ovid MEDLINE(R) ALL <1946 to July 15, 2022> MEDLINE: Medical Literature Analysis and Retrieval System Online

#	Query	Results from 18 July 2022
1	(child* or pre-school or preschool or paediatric* or pediatric*).mp. [mp=title, abstract, original title, name of substance word, subject heading word, floating sub-heading word, keyword heading word, organism supplementary concept word, protocol supplementary concept word, rare disease supplementary concept word, unique identifier, synonyms]	2,761,927
2	exp Child/	2,088,863
3	1 or 2	2,761,927
4	(temperat* or climat* or weather or (natur* adj3 environment*) or (built adj3 environment*) or (modified adj3 environment*) or (physical adj3 environment*) or (obesogenic adj3 environment*) or (environment* adj3 design) or walkability or (street adj3 connect*)).mp. [mp=title, abstract, original title, name of substance word, subject heading word, floating sub-heading word, keyword heading word, organism supplementary concept word, protocol supplementary concept word, rare disease supplementary concept word, unique identifier, synonyms]	1,166,755
5	exp Temperature/	442,764
6	exp Built Environment/	1,163
7	4 or 5 or 6	1,184,083
8	((physical adj3 activit*) or activit* or inactivit* or exercis*).mp. [mp=title, abstract, original title, name of substance word, subject heading word, floating sub-heading word, keyword heading word, organism supplementary concept word, protocol supplementary concept word, rare disease supplementary concept word, unique identifier, synonyms]	3,936,266
9	exp Exercise/	233,857
10	8 or 9	3,981,060
11	(overweight or obesity or BMI or (body mass adj3 index) or (paediatric adj3 obesity) or (pediatric adj3 obesity)).mp. [mp=title, abstract, original title, name of substance word, subject heading word, floating sub-heading word, keyword heading word, organism supplementary concept word, protocol supplementary concept word, rare disease supplementary concept word, unique identifier, synonyms]	607,765
12	exp Obesity/ or exp Overweight/	257,631
13	11 or 12	609,811
14	3 and 7 and 10 and 13	986
15	limit 14 to (English language and yr="2017 - 2022")	320
16	limit 15 to "child (6 to 12 years)"	207
	FINAL NUMBER OF ARTICLES	207

**Table 6:** Cochrane search Search Name: Cochrane January 2017 to July 2022 Date Run: July 18, 2022 14:49:15; 120 results without reviews and protocols

#	Search	Hits
1	(child*) or “pre-school” or (preschool) or (paediatric*) or (pediatric)	212,000
2	MeSH descriptor: [Child] explode all trees	61,542
3	#1 OR #2	212,000
4	(temperat*) or (climat*) or (weather) or (natur* NEAR/2 environment*) or (built NEAR/2 environment*) or (modified NEAR/2 environment*) or (physical NEAR/2 environment*) or (obesogenic NEAR/2 environment*) or (environment* NEAR/2 design) or (walkability) or (street NEAR/2 connect*)	28,920
5	MeSH descriptor: [Temperature] explode all trees	4,640
6	MeSH descriptor: [Built Environment] explode all trees	14
7	#4 OR #5 OR #6	29,018
8	(physical NEAR/2 activit*) or (activit*) or (inactivit*) or (exercis*)	281,873
9	MeSH descriptor: [Exercise] explode all trees	28,501
10	#8 OR #9	284,089
11	(overweight) or (obesity) or (BMI) or (body mass NEAR/2 index) or (paediatric NEAR/2 obesity) or (pediatric NEAR/2 obesity)	99,633
12	MeSH descriptor: [Overweight] explode all trees	18,989
13	MeSH descriptor: [Obesity] explode all trees	15,872
14	#11 OR #12 OR #13	99,714
15	#3 AND #7 AND #10 AND #14	311
16	#3 AND #7 AND #10 AND #14 with Cochrane Library publication date Between January 2017 and July 2022	209
17	#3 AND #7 AND #10 AND #14 with Cochrane Library publication date Between January 2017 and July 2022, in Cochrane Protocols, Trials, Clinical Answers, Editorials, Special Collections	135
18	#3 AND #7 AND #10 AND #14 with Cochrane Library publication date Between January 2017 and July 2022, in Trials, Clinical Answers, Editorials, Special Collections	120
	FINAL NUMBER OF ARTICLES	120

**Table 7:** Web of Science search Search date: July 18, 2022

Search	Articles retrieved
(((TS=((child*) or (pre-school) or (preschool) or (paediatric*) or (pediatric*))) AND TS=((temperat*) or (climat*) or (weather) or (natur* NEAR/2 environment*) or (built NEAR/2 environment*) or (modified NEAR/2 environment*) or (physical NEAR/2 environment*) or (obesogenic NEAR/2 environment*) or (environment* NEAR/2 design) or (walkability) or (street NEAR/2 connect*) )) AND TS=((physical NEAR/2 activit*) or (activit*) or (inactivit*) or (exercis*))) AND TS=((overweight) or (obesity) or (BMI) or (body mass NEAR/2 index) or (paediatric NEAR/2 obesity) or (pediatric NEAR/2 obesity))	1,776
Limits Applied	
Time limit (2017-2022)	742
English language and time limit	719
FINAL NUMBER OF ARTICLES	719

Appendix B: detailed search methodology

**Table 8:** The literature search was informed by these steps

Steps	
The Scoping Phase	It was conducted to assess the extent of available literature. At this stage, it was not realized that literature on the natural environment would be scarce. Initially, during this phase, a broader childhood age range of 2-18 years was considered. For children below six years of age, the empowering role of parents or caretakers could undermine the child’s independence and thus affect the utilization of neighbourhood resources by children. Whereas children above 12 years were considered to be less affected directly by their neighbourhood environmental factors because of their newly gained independence and ability to access facilities beyond the confines of their home neighbourhoods. Therefore, it was subsequently decided that to answer the research question, a child age range of 6-12 years was most suitable.
Search Strategy Development	This stage involved the development of search terms and inputting the search into different databases. Search terms related to the key concepts of all three review questions were identified. The terms ‘natural environment’, ‘temperature’, ‘built environment’, ‘overweight’, ‘obesity’, and ‘physical activity’ were included to retrieve all relevant articles. The following three databases were identified as relevant to the topic: MEDLINE using Ovid, Web of Science using Clarivate, and Cochrane using the Wiley-Blackwell interface. The search strategy was built in consultation with the London School of Hygiene and Tropical Medicine (LSHTM) library team. Thereafter, the final search was conducted on July 18, 2022, in the aforementioned databases. It comprised a keyword search for all databases combined with a controlled vocabulary search for databases that offered it, that is, MEDLINE and Cochrane. A total of 1046 articles were retrieved by our search. A total of 64 duplications were removed, first, using EndNote 20 version 20.4 (45 articles), and then using the software, Rayyan for the screening process (19 articles), leaving a total of 982 articles for title and abstract screening.
Study Selection (Screening)	Title and Keyword Screening: Title screening was based on eligibility criteria. The mention of the ‘adult’-only population in the title led to the exclusion of the study. Literature studying the pre-defined natural or built environmental exposures of interest was included at this stage, while research studying otherwise was excluded. Articles studying an outcome other than obesity, such as ‘physical activity’, ‘weight behaviours’, ‘diet’, etc. were excluded. Similarly, articles studying solely the dietary factors of obesity were excluded. If ‘physical activity’ was an outcome additional to obesity, it was included although evidence related to obesity only was assimilated in the review. Qualitative studies, and secondary research including literature reviews and overview of systematic reviews, were excluded. For the exclusion of qualitative studies, words like ‘qualitative’, ‘explore’, perspectives’, ‘perceived’, ‘barriers’, experiences’, etc. and alternative forms of these words in the title, aided their exclusion. The exclusion of reviews was aided by the Rayyan software in which words like ‘review’, and ‘systematic review’ were flagged in red by default. The search strategy was created to retrieve the articles from within the time frame selected. In instances where any of the above PICOST criteria were not clear on reading the title, abstract keywords highlighted by default in the Rayyan interface were also read. It resulted in 44 articles. Abstract screening: A similar methodology was applied to this stage as in the above. A full read of the abstracts allowed for greater clarity of PICOST eligibility criteria whereby 22 of 44 articles were excluded. Full-text screening: Thereafter, 22 potential articles remained, full texts of which were retrieved and read. Of these, 11 were excluded as they did not conform to the PICOST eligibility criteria [32-42]. For example, some studies were excluded because the population consisted of a wider childhood age range not conforming to our age group of interest despite the allowed margin of one year [34,39,42]. Others were excluded as they especially studied children having a higher risk of obesity [35,40]. Exposure to school environment, either within-school environment or the neighbourhood around schools, was studied in three articles; hence, these were excluded [32,36,38]. The studies by Barnett et al., Lin, and Saelens et al. were excluded because they did not study directly the association of built environment with obesity as an outcome [33,37,41]. Barnett et al. combined walkability and traffic safety into a composite exposure variable to generate typologies [33], whilst Saelens et al. combined physical activity and diet [41]. In both studies, individual components were not analysed, hence association with exposure of interests eligible to this review could not be drawn and these studies were excluded [41]. Lin discussed BMI as the exposure variable and physical activity as the outcome or dependent variable, which did not conform to our criteria of exposure and outcome, leading to its exclusion [37]. Note that Kimberly Daniels authored two different studies both published in 2021. One is a longitudinal study [45] and the other is cross-sectional [46]. Both are included in this review. In addition to the results of the search strategy, random snowballing identified six titles of relevance [43,83-87]. Amongst these, one was found relevant to this review topic as well as conforming to eligibility criteria, therefore it was included [43]. With regard to the temperature arm of the review, seven articles of potential relevance were considered. Five were excluded from title screening. Of these five, one studied the association of weather conditions in adults [56], two studied the association of weather with physical activity and not with obesity [57,58], the fourth, the outcome was blood pressure [59], while in the fifth, the outcome was mental well-being [60]. The full text was read for the remaining two. One was excluded as it did not study childhood obesity as the outcome [61]. The last was a review article and was therefore excluded [17]. The articles included in this review article by Jia and Dai et al. were studied for potential inclusion. Literature had been searched up to 2018, identifying six articles. None of these had been published in the last five years (2017-2022). While three were published in the last 10 years and the remaining even earlier. Of the three in the past 10 years, only two articles studied weather conditions, including temperature [16,62] as the exposure and the outcome was physical activity, not obesity. Since no articles met the eligibility criteria in the past five years, the search of literature related to the natural environment was extended to the past 10 years. This new scoping search identified 10 articles of potential relevance. In three, the population was adults, not children [63-65], whereas in six articles, the outcome was not obesity [16,58,66-69]. In the last article, neither the population coincided with the childhood age range nor obesity was the studied outcome [70]. Hence, no eligible literature was found even in the extended search for the past 10 years. In conclusion, as per the search strategy, no literature was found in the past five years that studied the relationship between the natural environment (temperature) and childhood (6-12 years) obesity. In the final review, 12 articles were included that studied the association of the built environment with overweight or obesity in children aged 6-12 years published from 2017 onward [43-54].
Data Extraction	To collect the data from the 12 included studies, a standardized data extraction sheet was developed in Microsoft Excel for Mac (2019) version 16.63.1. Information was retrieved on indexing, bibliographic details, population age and characteristics, methods including study design, results, strengths and limitations of the studies. Important information extracted is presented in Tables 1-4.
